# Combination therapy with vemurafenib (PLX4032/RG7204) and metformin in melanoma cell lines with distinct driver mutations

**DOI:** 10.1186/1479-5876-9-76

**Published:** 2011-05-24

**Authors:** Franziska Niehr, Erika von Euw, Narsis Attar, Deliang Guo, Doug Matsunaga, Hooman Sazegar, Charles Ng, John A Glaspy, Juan A Recio, Roger S Lo, Paul S Mischel, Begonya Comin-Anduix, Antoni Ribas

**Affiliations:** 1 Department of Medicine, Division of Hematology/Oncology, University of California Los Angeles (UCLA), Los Angeles, CA, USA; 2 Department of Pathology and Laboratory Medicine, UCLA, Los Angeles, CA, USA; 3 Jonsson Comprehensive Cancer Center at UCLA, Los Angeles, CA, USA; 4 Vall d'Hebron Research Institute, Barcelona, Spain; 5 Department of Medicine, Division of Dermatology, UCLA, Los Angeles, CA, USA; 6 Department of Surgery, Division of Surgical Oncology, UCLA, Los Angeles, CA, USA

## Abstract

**Background:**

A molecular linkage between the MAPK and the LKB1-AMPK energy sensor pathways suggests that combined MAPK oncogene inhibition and metabolic modulation of AMPK would be more effective than either manipulation alone in melanoma cell lines.

**Materials and methods:**

The combination of the BRAF inhibitor vemurafenib (formerly PLX4032) and metformin were tested against a panel of human melanoma cell lines with defined BRAF and NRAS mutations for effects on viability, cell cycle and apoptosis. Signaling molecules in the MAPK, PI3K-AKT and LKB1-AMPK pathways were studied by Western blot.

**Results:**

Single agent metformin inhibited proliferation in 12 out of 19 cell lines irrespective of the BRAF mutation status, but in one NRAS^Q61K ^mutant cell line it powerfully stimulated cell growth. Synergistic anti-proliferative effects of the combination of metformin with vemurafenib were observed in 6 out of 11 BRAF^V600E ^mutants, including highly synergistic effects in two BRAF^V600E ^mutant melanoma cell lines. Antagonistic effects were noted in some cell lines, in particular in BRAF^V600E ^mutant cell lines resistant to single agent vemurafenib. Seven out of 8 BRAF wild type cell lines showed marginally synergistic anti-proliferative effects with the combination, and one cell line had highly antagonistic effects with the combination. The differential effects were not dependent on the sensitivity to each drug alone, effects on cell cycle or signaling pathways.

**Conclusions:**

The combination of vemurafenib and metformin tended to have stronger anti-proliferative effects on BRAF^V600E ^mutant cell lines. However, determinants of vemurafenib and metformin synergism or antagonism need to be understood with greater detail before any potential clinical utility of this combination.

## Introduction

Mutually exclusive mutations in *NRAS *and *BRAF *provide oncogenic driver signals in melanoma of skin origin through the constitutive activation of the mitogen-activated protein kinase (MAPK) pathway [1-3]. The clinical relevance of blocking these driver mutations is highlighted by the targeted inhibition of BRAF^V600E ^with class I RAF inhibitors like vemurafenib (formerly PLX4032 or RG7204) or GSK2118436, which led to unprecedented high response rates in patients with metastatic melanoma [4,5]. Unfortunately, most responses are transient in part due to the development of secondary mutations in NRAS [6], increased expression of the cancer Osaka thyroid (COT, also known as MAP3K8) kinase [7], or more commonly the upregulation of receptor tyrosine kinases (RTKs) like the platelet-derived growth factor receptor beta (PDGFRβ) [6] or the insulin-like growth factor-1 receptor (IGF1R) [8].

Recent research suggests that there is a molecular linkage between the MAPK pathway and the LKB1-AMPK energy sensor pathway [9,10]. The liver kinase B1 (LKB1) is a serine/threonine kinase that functions as a tumor suppressor gene and is inactivated in Peutz-Jeghers syndrome. LKB1, together with low energy conditions, activates the AMP-activated protein kinase (AMPK), which results in inhibition of cell growth and proliferation. LKB1 can be phosphorylated by ERK (at Ser325) and p90^RSK ^(at Ser428), which compromise its ability to bind and activate AMPK. In BRAF^V600E ^mutant melanoma cells there is an uncoupling of the LKB1-AMPK complex [9,10]. These data suggest that AMPK can no longer be phosphorylated and therefore activated by LKB1, resulting in AMPK being unable to inhibit cell growth, proliferation and survival. This uncoupling of the LKB1-AMPK complex allows BRAF^V600E ^oncogene-driven cancer cells to become resistant to energy stress and avoid apoptosis [9].

AMPK functions as a master cellular energy sensor that is activated by metabolic stress, which results in an increase of the cellular AMP/ATP ratio either by inhibiting ATP synthesis (e.g. due to ischemia or hypoxia) or accelerating ATP consumption (e.g. due to muscle contraction). Once activated by phosphorylation at Thr172, AMPK leads to the inhibition of ATP-consuming processes like gluconeogenesis and fatty acid synthesis, and stimulates ATP generating processes like fatty acid oxidation [11]. AMPK inhibits fatty acid synthesis via phosphorylation and therefore inactivation of the ACC1 isoform of acetyl CoA carboxylase (ACC) [12] or suppression of lipogenic gene expression, including *ACC1 *and *fatty acid synthase*, and increase of fatty acid oxidation by phosphorylation and inactivation of ACC2 [13]. A recent manuscript reported on the direct antitumor effects of modulating AMPK in two melanoma cell lines, one with a BRAF mutation and another with an NRAS mutation [14]. This study suggested that AMPK may have a role as a negative regulator and suppressor of malignant melanoma cell growth, further promoting evidence of expanding the testing of the role of AMPK activation for melanoma treatment.

Metformin is a derivative of guanidine, one of the active ingredients (along with the isoprenyl guanidine derivative, galegine) in the ancient herbal remedy French Lilac (Galega officinalis). Metformin has been used to treat type-2 diabetes for over 50 years. It reduces blood glucose levels mainly through inhibition of hepatic gluconeogenesis. In order to enter the cell, metformin and its analogue phenformin need the organic cation transporter-1 (OCT1). There it can inhibit mitochondrial ATP production and thus activate AMPK indirectly by increasing the cellular AMP/ATP ratio [11]. The deletion of LKB1 in mice abolished the effect of metformin on AMPK activity and blood glucose levels, establishing the role of LKB1 as the main kinase that mediates AMPK activation upon exposure to metformin [15].

In this manuscript we tested the hypothesis that combination of BRAF oncogene inhibition and metabolic modulation of AMPK would be more effective than either manipulation alone in arresting melanoma cell proliferation. We tested a combination of vemurafenib and metformin in a panel of melanoma cell lines with defined BRAF and NRAS mutations. The range of concentrations for both agents was chosen based on prior reports on the single agent activity of each one of them [14,16,17]. In our studies, the combination of vemurafenib and metformin synergistically inhibited proliferation in a subset of human melanoma cell lines and induced cell cycle arrest or apoptosis, but the differential effects in cell lines was not fully explained by the modulation of the MAPK and AMPK-LKB1 pathways.

## Materials and methods

### Reagents

Vemurafenib (also known as PLX4032, RG7204 or RO5185426) was obtained from Dr. Gideon Bollag under a materials transfer agreement (MTA) with Plexxikon (Berkeley, CA). The compound was dissolved in dimethyl sulfoxide (DMSO, Sigma-Aldrich, St. Louis, MO) to a concentration of 100 mM and additionally in RPMI 1640 media (Mediatech Inc., Manassas, VA) to a working stock concentration of 100 μM. Metformin (also known as 1,1-dimethylbiguanide hydrochloride) was purchased from Sigma-Aldrich and dissolved in RPMI media to a working stock concentration of 200 mM. Both stock solutions were stored at 4°C for up to 1 week.

### Cell culture

Human melanoma cell lines from the M series were derived from patient's biopsies under UCLA IRB approval #02-08-067 and have been previously described [16]. SKMEL28, WM1366, SBCL2 and SKMEL173 were obtained from American Type Culture Collection (Rockville, MD). All cell lines were mycoplasma free when periodically tested with the Mycoalert Mycoplasma Detection Kit (Lonza, Rockland, ME).

### Cell proliferation assays

Melanoma cell lines were treated in triplicate with vemurafenib, metformin or parallel vehicle control at the given concentrations for 72 hours. Cell viability was measured using a tetrazolium compound [3-(4,5-dimethylthiazol-2-yl)-5-(3-carboxymethoxyphenyl)-2-(4-sulfophenyl)-2H-tetrazolium (MTS)]-based colorimetric cell proliferation assay (Promega, Madison, WI) as previously described [16].

### Cell cycle analysis

Cells were treated with different concentrations of vemurafenib, metformin, the combination or parallel vehicle control for 24 hours, fixed with Cytofix/Cytoperm (BD Biosciences, San Jose, CA), washed with Perm/Wash (BD Biosciences), and then resuspended in sterile PBS containing 0.5 μg/mL DAPI (Sigma-Aldrich). Flow cytometry analyses for this and other experiments was performed in an LSR-II (BD Biosciences) and data was analyzed using FlowJo (Tree Star Inc, Asland, OR).

### Apoptosis analysis

Cells were plated in 6-well plates and treated with DMSO, vemurafenib, metformin, the combination or 1 μM staurosporine as a positive control. After 72 hours apoptosis analysis was performed using the FITC Annexin V Apoptosis Detection Kit (BD Biosciences) following the manufacturer's instructions and analyzed by flow cytometry.

### Phospho-flow staining

Staining of phosphorylated AKT (p-AKT Thr308) in melanoma cells exposed to DMSO, vemurafenib, metformin or the combination for 24 hours was performed in permeabilized cells using the phosphoflow technique as previously described [18] and analyzed by flow cytometry.

### Western Blotting

Western blotting was performed as previously described [19]. Primary antibodies included pCRAF (Ser289/296/301), CRAF, pMEK1/2 (Ser217/221), MEK1/2, pERK1/2 (Thr202/Tyr204, Thr185/Tyr187), ERK1/2, pLKB1 (Ser428), LKB1, pAMPKα (Thr172), AMPKα, pACC (Ser79), ACC, pS6K (Thr389), S6K, pS6 (Ser235/236), S6, and β-actin (all from Cell Signaling Technology, Danvers, MA). The immunoreactivity was revealed by use of Pierce Super Signal West Pico Chemiluminescent Substrate (Thermo Scientific, Rockford, IL).

### Low glucose experiments

Cells were plated in standard high glucose RPMI media (3000 mg/L). After 24 hours, media was aspirated and low glucose media was added to the cells for at least 4 hours before treatment. Low glucose media consisted of RPMI media without glucose, supplemented with L-glutamine (Mediatech), to which 1000 mg/L D-glucose (Sigma-Aldrich), 10% FBS and 1% PSF were added. Cells were treated with different concentrations of DMSO, vemurafenib, metformin or the combination in low glucose media. For incubations longer than 24 hours, drugs were added freshly every day.

### Statistical Analysis

To determine synergistic, additive, or antagonistic effects of the drug combinations we used the combination index method of Chou and Talalay [20] using the CalcuSyn software (version 2.0 Biosoft, Cambridge, UK). This method takes into account both potency [median dose (Dm) or IC_50_] and the shape of the dose-effect curve (the *m *value) to calculate the combination index (CI). A CI equal to 1 indicates an additive effect; a CI less than 1 indicates synergy. With the use of CalcuSyn software, synergy is further refined as synergism (CI = 0.3-0.7), strong synergism (CI = 0.1-0.3), and very strong synergism (CI < 0.1).

## Results

### Cell growth inhibition with single agent vemurafenib or metformin

**Figure 1:**
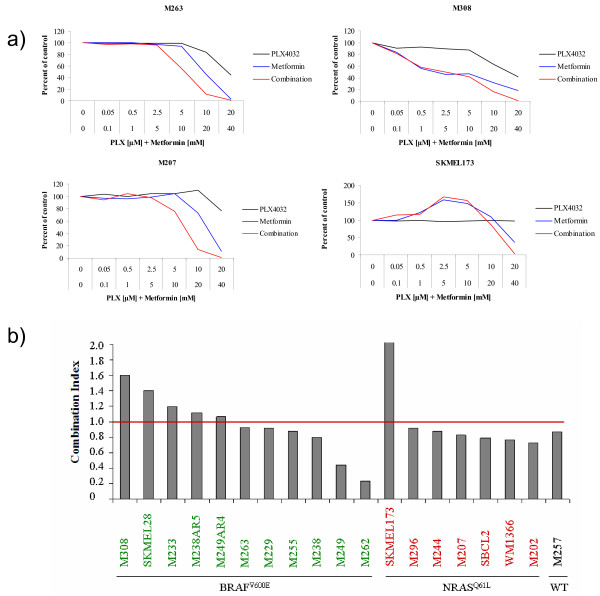
**Effects of vemurafenib, metformin or the combination on cell proliferation and viability**. a) Example of growth curves of BRAF^V600E ^and NRAS^Q61 ^mutant melanoma cell lines. BRAF^V600E ^cell lines included M263 (synergistic) and M308 (antagonistic). As representatives for NRAS^Q61 ^mutants M207 (synergistic) and SKMEL173 (antagonistic) are shown. The black line represents the data obtained with vemurafenib treatment, the blue line with metformin, and the red line with combination treatment. b) Combination index for the combination of 5 μM vemurafenib and 10 mM metformin of all melanoma cell lines tested. The green cell lines harbor BRAF^V600E^, red shows NRAS^Q61 ^mutant cell lines, and black the wild type cell line M257.

A panel of 19 melanoma cell lines with previously characterized [6,16] oncogenic alterations (Table 1) was first used to test the antitumor effects of single agent exposure to vemurafenib or metformin. This panel included previously established cell lines [16] and two cell lines that had been derived as sub-lines with acquired resistance to vemurafenib through continuous *in vitro *exposure to this agent [6]. These cell lines are representative of different oncogenic events in melanoma and include 10 cell lines with a BRAF (BRAF^V600E^) and 7 cell lines with an NRAS (NRAS^Q61L ^or NRAS^Q61K^) mutation [16]. M249-AR4, one of the *in vitro *derived sub-lines resistant to vemurafenib, harbors mutations both in BRAF^V600E ^and NRAS^Q61K ^[6]. One cell line, M257, is wild type for both oncogenes. In agreement with prior studies [6,16], single agent vemurafenib induced the expected growth inhibition restricted to BRAF^V600E ^mutant cell lines, while NRAS^Q61 ^or double wild type cell lines were resistant (Table 1 Figure 1a). In these studies, resistance to vemurafenib was defined by a 50% inhibitory concentrations (IC50) over 1 μM. Single agent metformin inhibited cell proliferation in 8 out of 11 BRAF^V600E^, and 3 out of 7 NRAS^Q61 ^mutant cell lines, as well as in the cell line wild type for both oncogenes, M257 (Table 1). Resistance to metformin was defined by IC50s over 20 mM. Of particular importance, single agent metformin led to a markedly increased and dose-dependent proliferation of one NRAS^Q61K ^mutant cell line, SKMEL173.

**Table 1:** Overview of differentially mutated melanoma cell lines tested.

Cell Line	BRAF/NRAS	*BRAF* Gene Copies	Other Oncogenic Events	IC50 for single agent vemurafenib	IC50 for single agent metformin
M257	Wild type	3	*CDKN2A *R80	*12.2 μM*	*16.4 mM*
M202	NRAS^Q61L^	2	*EGFR *amplification, *CDKN2A *^-/-^	*> 20 μM*	*25.3 mM*
M207	NRAS^Q61L^	2	*MITF *amplification, *PTEN *^-/+^	*> 20 μM*	*25.2 mM*
M244	NRAS^Q61L^	*NT*	*NT*	*19.9 μM*	*22.7 mM*
M296	NRAS^Q61L^	*NT*	*NT*	*19.4 μM*	*9.0 mM*
SKMEL173	NRAS^Q61K^	*NT*	*NT*	*> 20 μM*	*36.5 mM*
WM1366	NRAS^Q61L^	*NT*	*NT*	*> 20 μM*	*8.6 mM*
SBCL2	NRAS^Q61L^	*NT*	*NT*	*> 20 μM*	*2.9 mM*
M229	BRAF^V600E ^homozygous	4	*MITF *amplification, *AKT1 *amplification *PTEN *^-/+^	*0.3 μM*	*13.0 mM*
M233	BRAF^V600E ^heterozygous	3	*AKT1 *amplification, *CCND1 *amplification *EGFR *amplification, *CDKN2A *^-/-^, *PTEN *^-/-^	*> 20 μM*	*20.2 mM*
M238	BRAF^V600E ^heterozygous	2	*CDKN2A *^-/-^, *PTEN *^-/+^	*0.1 μM*	*12.5 mM*
M238-AR5	BRAF^V600E ^heterozygous	2	*CDKN2A *^-/-^, *PTEN *^-/+^, PDGFR1b over-expression	*> 20 μM*	*13.1 mM*
M249	BRAF^V600E ^heterozygous	3	MITF amplification, AKT2 amplification, *PTEN *^-/-^	*0.1 μM*	*9.2 mM*
M249-AR4	BRAF^V600E ^heterozygous NRAS^Q61K^	3	MITF amplification, AKT2 amplification, *PTEN *^-/-^	*> 20 μM*	*17.9 mM*
M255	BRAF^V600E ^heterozygous	2	*AKT2 *amplification, *CCND1 *amplification *EGFR *amplification, *CDKN2A *^-/-^	*8.8 μM*	*17.3 mM*
M262	BRAF^V600E ^homozygous	2	AKT1 E17K, AKT1 amplification *EGFR *amplification, *CDKN2A *^-/-^	*0.2 μM*	*20.5 mM*
M263	BRAF^V600E ^heterozygous	2	*CDKN2A *^-/+^	*19.9 μM*	*19.6 mM*
M308	BRAF^V600E ^heterozygous	3	*MITF *amplification *AKT2 *amplification, *EGFR *amplification *CDKN2A-/+*	*17.3 μM*	*6.0 mM*
SKMEL28	BRAF^V600E ^homozygous	2	*EGFR *P753S, *MITF *amplification *CCND1 *amplification, *CDKN2A *-/+, *PTEN *-/+	*1.5 μM*	*29.7 mM*

Legend: NT: not tested.

### Synergistic growth inhibition by the combination of vemurafenib and metformin in a subset of melanoma cell lines independent of the BRAF mutational status

After evaluating the response of the differentially mutated melanoma cells to each drug alone, both drugs were combined in constant ratios to each other. Among the 11 BRAF^V600E ^mutant cell lines, in 5 cell lines the combination was antagonistic and in 6 cell lines it was synergistic (Figure 1b). In two cell lines (M249, M262) the combination was highly synergistic. Of note, the two cell lines with acquired resistance to vemurafenib had antagonistic effects from combined vemurafenib and metformin. Among the 8 BRAF wild type cell lines, the combination was synergistic in 7, with the only one with antagonistic effects being SKMEL173, which already had increased proliferation with single agent metformin. However, none of the BRAF wild type cell lines displayed a highly synergistic growth-inhibitory response to combined vemurafenib and metformin exposure.

### The combination of vemurafenib and metformin inhibited cell cycle progression in a larger fraction of cell lines compared to single agent therapy

**Figure 2:**
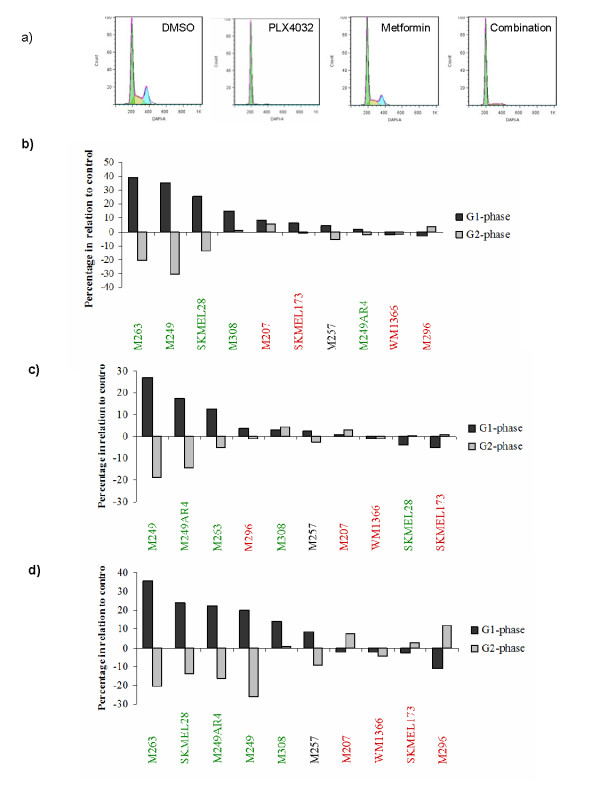
**Effects of single agent and combination therapy on cell cycle progression**. Melanoma cells were treated with 5 μM vemurafenib, 10 mM metformin, or the combination for 24 hours. Afterwards cell cycle analysis was performed using DAPI staining and analyzed by flow cytometry. a) Cell cycle analysis of M263 treated with DMSO, vemurafenib, metformin, and the combination. b) Cell cycle changes after single agent vemurafenib. c) Cell cycle changes after single agent metformin. d) Cell cycle changes after combination of vemurafenib and metformin. Columns indicate a ratio of change over baseline (DMSO treatment). BRAF^V600E ^mutants are presented in green, NRAS^Q61 ^in red, and the wild type M257 in black.

To further characterize the effects of the combination, 10 cell lines were chosen for further detailed analyses of cell signaling and proliferation (Table 2). They represented the major cell line groups based on oncogenic events and response to the combination of vemurafenib and metformin. Effects on cell cycle were studied using flow cytometry to assess changes in the G1, S and G2 phases of the cell cycle with either agent compared to DMSO (Figure 2a). In agreement with our prior studies [16], single agent vemurafenib inhibited the cell cycle with a G1 arrest in a BRAF^V600E^-restricted fashion, with an effect less pronounced in the naturally resistant M308 cell line (Figure 2b). There was no cell cycle arrest in the acquired resistant M249-AR4 cell line, consistent with the presence of a secondary mutation in NRAS^Q61K ^[6]. Single agent metformin also induced G1 arrest in three BRAF^V600E ^mutant cell lines, with no effects on the rest. The inhibition was independent of the sensitivity to metformin observed in proliferation assays (Figure 2c), suggesting that metformin may have antitumor effects independent of the cell cycle. The combination of vemurafenib and metformin inhibited cell cycle progression apparently by combining the growth arrest effects of each single agent in BRAF^V600E ^mutant cell lines, with an additional inhibitory effect against the wild type M257 cell line (Figure 2d). In two NRAS^Q61 ^mutant cell lines the combination induced a G2 arrest. Of note, the effects of single agent metformin or the combination with vemurafenib on the cell cycle were minimal in SKMEL173, the cell line that demonstrated enhanced proliferation with these treatments.

**Table 2:** BRAF^V600E ^and BRAF^WT ^cell lines used for detailed analysis.

Cell Line	BRAF/NRAS	Cell line characteristics
M249	BRAF^V600E^	Single agent vemurafenib: Highly sensitive Single agent metformin: Highly sensitive Combination: Slight synergy
M249-AR4	BRAF^V600E^ NRAS^Q61K^	Single agent vemurafenib: Resistant Single agent metformin: Intermediate sensitive Combination: Antagonistic
M263	BRAF^V600E^	Single agent vemurafenib: Resistant Single agent metformin: Intermediate sensitive Combination: Slight synergy
M308	BRAF^V600E^	Single agent vemurafenib: Resistant Single agent metformin: Highly sensitive Combination: Antagonistic
SKMEL28	BRAF^V600E^	Single agent vemurafenib: Intermediate sensitive Single agent metformin: Resistant Combination: Antagonistic
M207	NRAS^Q61L^	Single agent vemurafenib: Resistant Single agent metformin: Resistant Combination: Slight synergy
M296	NRAS^Q61L^	Single agent vemurafenib: Resistant Single agent metformin: Highly sensitive Combination: Slight synergy
SKMEL173	NRAS^Q61K^	Single agent vemurafenib: Resistant Single agent metformin: Resistant Combination: Highly antagonistic
WM1366	NRAS	Single agent vemurafenib: Resistant Single agent metformin: Highly sensitive Combination: Slight synergy
M257	Wild type	Single agent vemurafenib: Resistant Single agent metformin: Intermediate sensitive Combination: Slight synergy

Legend: Sensitivity to single agent vemurafenib: highly sensitive if IC50 < 1 μM; intermediate sensitive with IC50 1-10 μM; resistant if IC50 > 10 μM. Sensitivity to single agent metformin: highly sensitive if IC50 < 10 mM; intermediate sensitive with IC50 10-20 mM; resistant if IC50 > 20 mM. Sensitivity to the combination of vemurafenib and metformin: CI > 1 antagonistic; CI 1-0.3 slight synergy; CI < 0.3 strong synergy.

### Induction of apoptosis by vemurafenib, metformin or the combination

**Figure 3:**
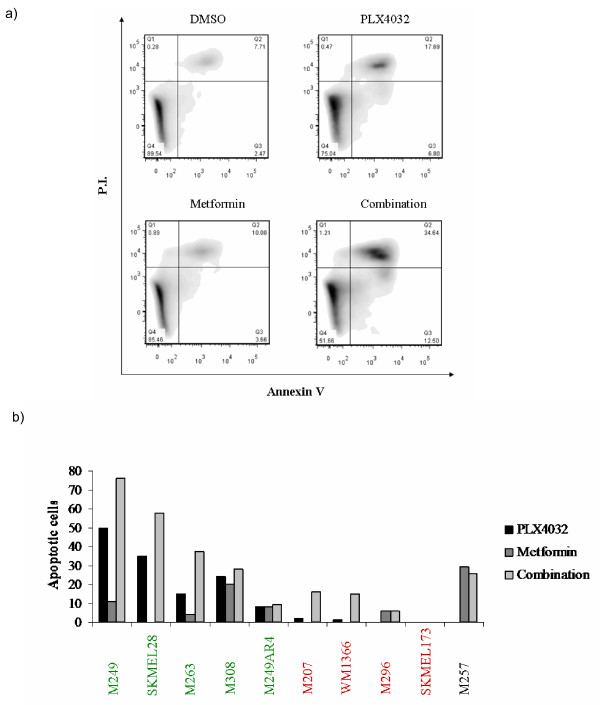
**Effects of single agent and combination therapy on apoptosis**. To test the cytotoxic effects of the different treatments, melanoma cells were treated for 72 hours with 5 μM vemurafenib, 10 mM metformin, or the combination. Cells were stained with Annexin V and PI and were analyzed by flow cytometry. a) Annexin/PI staining of M263 as a representative example. Upper left corner: DMSO, upper right corner: vemurafenib, lower left corner: metformin, lower right corner: Combination of vemurafenib and metformin. b) Early and late apoptotic cells after 72-hour treatment with the indicated agents. Percentages are shown in relation to the control, DMSO treated cells.

Effects on apoptotic cell death were studied by flow cytometry using co-staining with Annexin V and Propidium Iodide (Figure 3a). As expected [16], single agent vemurafenib induced significant levels of apoptosis in all the BRAF^V600E ^mutant cell lines that are sensitive to this agent, with a much less effect in the resistant ones (Figure 3b). Single agent metformin induced apoptotic effects only in the BRAF^V600E ^cell lines with strong (M249, M308) or moderate (M257) sensitivity to single agent metformin. In four out of six cell lines with synergistic characteristics, the effect of the combination was greater than the sum of each individual drug effect. In the rest there was either an effect due to the sum of each drug alone or no apoptotic effect at all. Cell lines with antagonistic characteristics showed no effect or an effect less than the sum of single agents. Interestingly, in the cell line SKMEL28 (antagonistic) the combination showed a greater apoptotic effect compared to either single drug treatment.

### Analysis of signal transduction changes with the combination of vemurafenib and metformin

**Figure 4:**
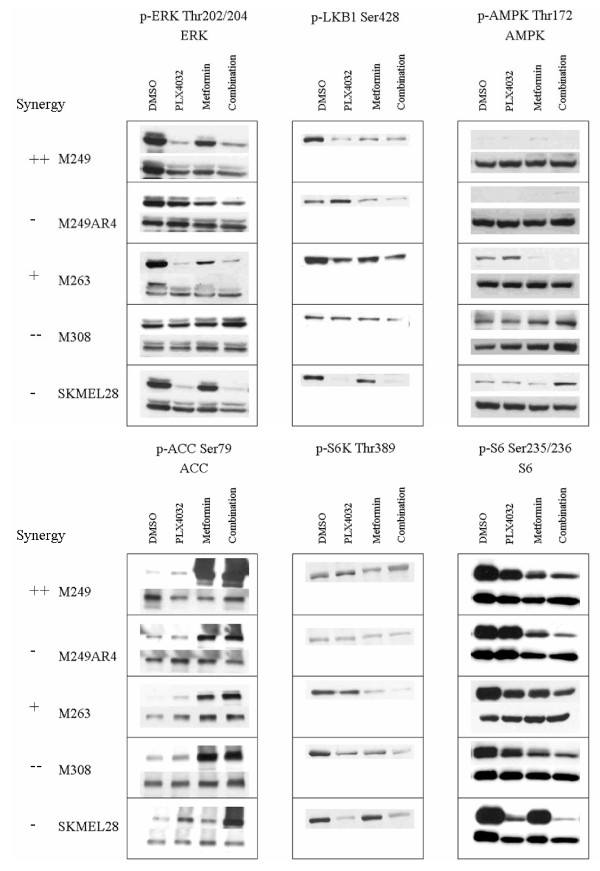
**Western blot analysis of different BRAF^V600E ^mutant cell lines**. Cells were exposed for 24 hours to 5 μM vemurafenib, 10 mM metformin, or the combination. Phosphorylation was analyzed with antibodies against specific phosphor-proteins compared to their total proteins. Synergistic characteristics of the cell lines are indicated as + (slight synergy), ++ (significant synergy), - (slight antagonism), and - - (significant antagonism).

We next analyzed differential signaling pathways blocked or induced by either drug treatment. An example of the complete Western blot analysis of two representative cell lines is included in Additional File 1 and a time course analysis in Additional File 2. A summary of the key findings focusing on the 24 hour time point in BRAF^V600E ^mutant cell lines is presented in Figure 4. At this time point, the effects on pERK and pLKB1 were mainly derived from single agent vemurafenib, where sensitive BRAF^V600E ^mutant cell lines had significant decrease with either single agent or combination containing vemurafenib. Single agent metformin had minimal effects on pERK or pLKB1, although in some cases (for example M249 and M263, Figure 4 and Additional File 1) there was a partial decrease in pERK. Of note, the acquired resistant M249-AR4 cell line had increased pLKB1 phosphorylation with vemurafenib, which decreased with the combination. The effects on pAMPK did not directly follow the effects on pLKB1 at the 24 hour time point, with the exception of SKMEL28 where the decrease in pLKB1 resulted in increase in pAMPK, which is consistent with published data [9,10]. Conversely, the effects on pACC were mainly derived from single agent metformin, where in most cell lines the increases in pACC were noted concordantly with single agent metformin and the combination with vemurafenib. The effects on p-p70 S6K were mainly dependent on the sensitivity of each cell line to single agent vemurafenib, where resistant cell lines had no decreased phosphorylation. There was little contribution of metformin, which was restricted to a further inhibition of p-p70 S6K in M263 with both single agent and combination metformin. Effects on pS6 were more variable with single agent vemurafenib or metformin. However, there was a trend towards decreased phosphorylation with the combination, which was notable in all tested cell lines. Overall, no single phosphorylated molecule change or combination allowed discerning the effects of the combination to provide synergy or antagonism.

**Figure 5:**
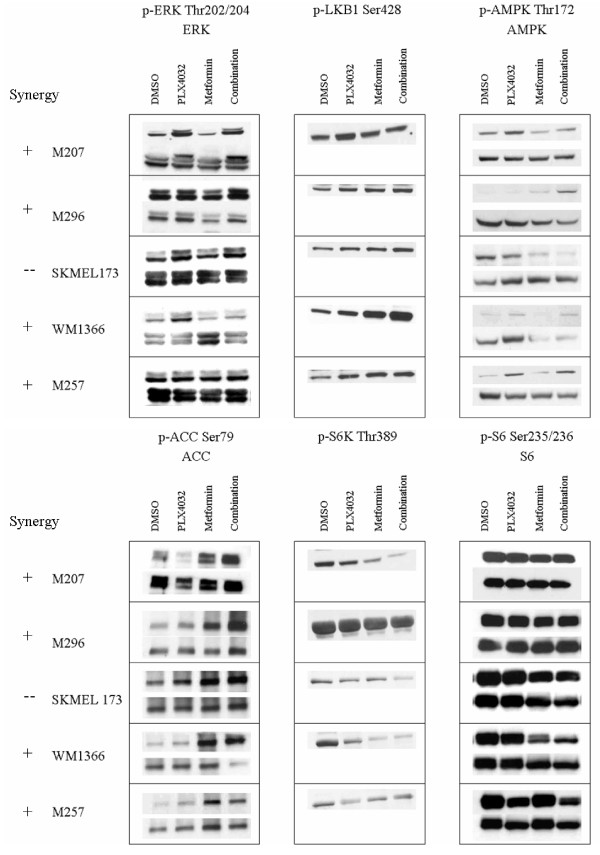
**Western blot analysis of different BRAF^WT ^cell lines**. Cells were exposed for 24 hours to 5 μM vemurafenib, 10 mM metformin, or the combination. Phosphorylation was analyzed with antibodies against specific phosphor-proteins compared to their total proteins. Synergistic characteristics of the cell lines are indicated as + (slight synergy), ++ (significant synergy), - (slight antagonism), and - - (significant antagonism).

In BRAF wild type cell lines, single agent vemurafenib or the combination tended to increase pERK as previously described [16], without clearly concordant changes in pLKB1 (Figure 5). The most remarkable effect of the combination on phosphorylated signaling changes that followed sensitivity was the divergent effects on pAMPK between the antagonistic cell line SKMEL173 and the rest of NRAS^Q61 ^mutant cell lines. However, this change may be cell line-dependent, as opposed to a marker of differential effects of the combination, since it was not apparent in the BRAF^V600E ^mutant cell lines with antagonistic effects of vemurafenib combined with metformin. As with the BRAF^V600E ^mutant cell lines, effects on pACC followed the effects of single agent metformin with little contribution of vemurafenib. The change in the phosphorylation of p70 S6K and S6 did not contribute much to elucidating the effects of this combination.

### Quantitative analysis of AKT phosphorylation

Given a lack of a clear correlation between sensitivity to the combination and signaling changes in the MAPK and LKB1-AMPK pathways, we used the quantitative phospho-flow assay to better explore potentially differential effects on AKT phosphorylation that may be missed with Western blot analysis. Vemurafenib, metformin or the combination resulted in decreased phosphorylation of AKT in BRAF^V600E ^and BRAF^WT ^cell lines (Additional File 3). Each drug and the combination inhibited phosphorylation of AKT. The phosphorylation of AKT was independent from the driver mutation (BRAF^V600E^, NRAS^Q61^, or wild type), or the sensitivity to single agents or the combination. In fact, the combination of the drugs did not demonstrate differential effects on pAKT compared to either drug alone.

### Similar results in low glucose and high glucose media conditions

Standard cell culture media is supplemented with glucose at concentrations that are much higher than physiological concentrations in tumors. It is possible that the high glucose concentrations in cell culture experiments would offset the effects of AMPK activation by metformin. Therefore, we performed experiments using low glucose media compared to standard high glucose-containing media. Side-by-side studies in the M263 cell line with high or low glucose media demonstrated that there was no change in cell proliferation in MTS assays, cell cycle effects or apoptosis by flow cytometry (data not shown). Western Blot analysis (Additional File 4a) and pAKT (data not shown) flow cytometry were performed and results compared to those observed with high glucose conditions. Again, low glucose conditions alone did not induce major differential effects. The only notable difference was treatment of M263 with metformin and the combination, which resulted in an increase of pAMPK levels in low glucose media compared to a decrease in high glucose media. Consistent with this, phosphorylation of ACC was elevated and pS6 inhibited. Additionally, similar comparison studies with the antagonistic SKMEL28 cell line showed that vemurafenib treatment resulted in a slight increase in pAMPK, which was not found in the high glucose concentrations (Additional File 4b). Overall, the effects of metformin or the combination with vemurafenib were not significantly different in low or high glucose media for any of these assays.

## Discussion

The description of a molecular linkage between the MAPK and the LKB1-AMPK pathway in BRAF^V600E ^mutant cells suggested that cells with this mutation may escape metabolic stress-induced checkpoints [10]. A corollary would be that simultaneous blocking of the driver BRAF^V600E ^oncogene, together with stimulation of AMPK, may result in synergistic antitumor activity. This could be achieved with a combination of vemurafenib with metformin. We had anticipated that the combination would have synergistic effects only in cell lines that carry the BRAF^V600E ^mutation. The reactivation of AMPK brought by the inhibition of oncogenic BRAF^V600E ^with vemurafenib, together with the stimulation of AMPK with metformin, may co-operate to induce cell death in cells with this oncogenic driver mutation. However, this combination also showed similar effects in some BRAF wild type cells. Synergistic effects of the combination were observed in 6 out of 11 BRAF^V600E ^mutants, 6 out of 7 NRAS^Q61 ^mutants, and the cell line wild type for both oncogenes. This was not dependent on the sensitivity to each drug alone. However, we found highly synergistic activity of the combination only in a subset of BRAF^V600E ^mutant cell lines and none of the BRAF wild type cells. Interestingly, single agent metformin treatment in the NRAS^Q61K ^mutant SKMEL173 resulted in increased proliferation, which could not be explained by a differential effect of this agent on AMPK or the PI3K/AKT pathway. In addition, some of the BRAF^V600E ^and NRAS^Q61 ^mutants displayed antagonistic effects with the combination of vemurafenib and metformin.

The finding of some cell lines having divergent effects, together with the lack of predictive markers for either effect, raises serious concerns about the potential clinical use of metformin alone or in combination with vemurafenib for its hypothesized potential benefits in stimulating AMPK in melanoma cells. Of note, the two BRAF^V600E ^mutant cell lines with acquired resistance to vemurafenib had antagonistic effects with the combination with metformin. This suggests that the addition of metformin is unlikely to be of benefit in patients progressing on single agent vemurafenib.

We explored changes in phospho-protein levels as means to understand the differential effects of this combination in this panel of cell lines. In our studies a decrease of pERK after treatment with vemurafenib correlated well with a decrease in LKB1 phosphorylation. Dephosphorylated LKB1 is able to phosphorylate and activate AMPK. This correlation could not be seen after 24 hours, even though time-course experiments showed that pAMPK levels changed earlier (at 4 hours). Interestingly, even though pAMPK levels at 24 hours did not correlate well, pACC levels were always increased when pERK was inhibited. Therefore, effects of vemurafenib blocking oncogenic BRAF^V600E ^signaling are in accordance with their predicted effects based on prior studies [9,10]. An interesting observation is that in the BRAF^V600E ^mutant cell lines with highest sensitivity to the combination there was evidence of a pERK decrease after exposure to metformin. Therefore, it is possible that the synergistic effects of vemurafenib and metformin in these cell lines would be due to a more significant blockade of oncogenic MAPK signaling. However, the fact that single agent vemurafenib results in apparently complete inhibition of pERK by Western Blot in cell lines sensitive to this agent makes it difficult to analyze if the addition of metformin has an even stronger pERK inhibiting effect. The observation of strong correlation between pERK and pACC increase suggests that some of the effects of the combination may be related to modulation of lipogenesis [19].

The exact mechanism by which metformin activates AMPK, and if AMPK is the primary therapeutic target of metformin, is a matter of debate. One issue that comes up is the relatively high concentrations of the drug (1-20 mM) that are required for AMPK activation in cultured cells. Estimated concentrations in human plasma after a standard therapeutic dose of around 30 mg/kg range from 10-40 μM. An explanation for this could be the low level or lack of expression of the transporter OCT1 in many cultured cell lines [21]. Other metabolic regulators such as resveratrol, berberine and thiazolidinediones also inhibit mitochondrial ATP production and thus activate AMPK indirectly, although these do not require OCT1. Surprisingly, single agent metformin inhibited phosphorylation of ERK in some BRAF^V600E ^and NRAS^Q61 ^mutant melanoma cell lines. A time course experiment with M263 showed that this decrease was observed 8 hours after treatment. Since the metformin-induced increase in pACC was noted earlier, it suggests that the inhibitory effect of metformin on the MAPK pathway may be mediated by a feedback mechanism. Interestingly, pACC was elevated in all cell lines, indicating that AMPK activity must have been increased by metformin, since AMPK is responsible for ACC phosphorylation. ACC phosphorylation was dependent on the sensitivity to the drug, with those sensitive showing the strongest increase in pACC. Of note, research by Guo *et al*. [19] with AICAR showed that the anti-growth properties of this AMPK activator are not fully mediated through inhibition of mTORC1 signaling, but rather through inhibition of cholesterol and fatty acid synthesis by inhibition of ACC and HMG-CoA.

## Conclusions

Our studies provide evidence that the combination of an inhibitor of the MAPK pathway with an AMPK activator has synergistic antitumor effects in some BRAF^V600E ^and BRAF^WT ^cell lines, including NRAS^Q61 ^mutants. However, the differential effects of this combination could not be attributed to known oncogenic mutations nor differential modulation of MAPK, PI3K/AKT or LKB1-AMPK signaling pathways. In particular, the combination did not reverse natural or acquired resistance to vemurafenib in BRAF^V600E ^mutant cell lines, and the effects on BRAF^WT ^cell lines could not be explained by a unified effect on the signaling pathways studied by us. Given the observation of metformin paradoxically stimulating the growth of one cell line in our panel, and the antagonistic effects of the combination of metformin and vemurafenib in several cell lines, the combination of vemurafenib and metformin should not be considered clinically until a more detailed understanding of their differential effects is generated.

## Abbreviations

acetyl CoA carboxylase (ACC); AMP-activated protein kinase (AMPK); cancer Osaka thyroid (COT); combination index (CI); dimethyl sulfoxide (DMSO); 50% inhibitory concentrations (IC50); insulin-like growth factor-1 receptor (IGF1R); liver kinase B1 (LKB1); materials transfer agreement (MTA); tetrazolium compound [3-(4,5-dimethylthiazol-2-yl)-5-(3-carboxymethoxyphenyl)-2-(4-sulfophenyl)-2H-tetrazolium (MTS); organic cation transporter-1 (OCT1); phosphorylated Erk (pErk); platelet-derived growth factor receptor beta (PDGFRβ); receptor tyrosine kinases (RTKs)

## Competing interests

Antoni Ribas has received honoraria from Roche-Genentech, the maker of vemurafenib.

## Authors' contributions

FN, BC-A and AR planned the research. FN performed the majority of the experiments. She received help from EvE, NA and DG on the performance of Western Blot analysis, from DG on the use of metformin, from DM, HS and CN for the care of melanoma cell lines and cell viability studies and from BC-A for the optimization and conduct of cell viability, cell cycle, apoptosis and phosphoflow studies: JAG, JAR, RSL and PSM helped focus the research plan and discussed the proposed experiments, as well as provided interpretation of the emerging results and the studies that should follow. FN and AR wrote the manuscript, with significant contributions and edits from JAR, RSL and PSM. All authors read and approved the final manuscript.

## Supplementary Material

Additional file 1 **Western blot analysis of two cell lines with synergy with the combination**. To analyze the phospho-protein signaling events triggered by exposure to vemurafenib and metformin, cell lines were treated with DMSO, vemurafenib (5 μM), and metformin (10 mM), either singly or in combination, for 24 hours. Protein phosphorylation was examined by Western Blot analysis and phospho-specific flow cytometry. Blotting for total proteins and β-actin was used as a loading control. Phosphorylation was analyzed with antibodies against pRAF, pMEK, pERK, pLKB1, pAMPKα, pACC, pS6K, pS6, and their total proteins. a) BRAF^V600E ^mutant cell line M263; b) NRAS^Q61L ^mutant cell line M207. Click here for file

Additional file 2 **Time course analysis of signaling in two BRAFV600E mutant cell lines with different response to the combination**. Cells were treated for 2, 4, and 24 hours with 5 μM vemurafenib, 10 mM metformin, or the combination. a) M263, in which the combination showed slight synergistic effects in proliferation assays; b) SKMEL28, in which the combination had slight antagonistic effects in proliferation assays. Click here for file

Additional file 3 **Phospho-specific flow cytometry for p-Akt Thr308**. Cells were treated with vemurafenib (5 μM), metformin (10 mM) or the combination for 24 hours, and intracellularly stained with a pAKT Thr308 antibody. Percentages shown are in relation to DMSO controls and were analyzed by flow cytometry. Click here for file

Additional file 4 **Time-course Western blot analysis of the BRAF^V600E ^mutant cell lines M263 (top) and SKMEL28 (bottom) in low glucose media**. For these studies, the BRAF^V600E ^mutant cell lines M263, resistant to vemurafenib and sensitive to metformin, and SKMEL28, resistant to vemurafenib and metformin, were treated with agents dissolved in low glucose media (1000 mg/L) instead of normal RPMI (3000 mg/L) and analyzed at 4, 8 or 24 hours. Click here for file
