# Supplementary figures and images for: Combination therapy with vemurafenib (PLX4032/RG7204) and metformin in melanoma cell lines with distinct driver mutations
Source: J Transl Med. 2011 May 24;9:76. doi: 10.1186/1479-5876-9-76 (PMC3152784; doi:10.1186/1479-5876-9-76)

# Supplemental Figure 1

## a. M263 (BRAF mt)

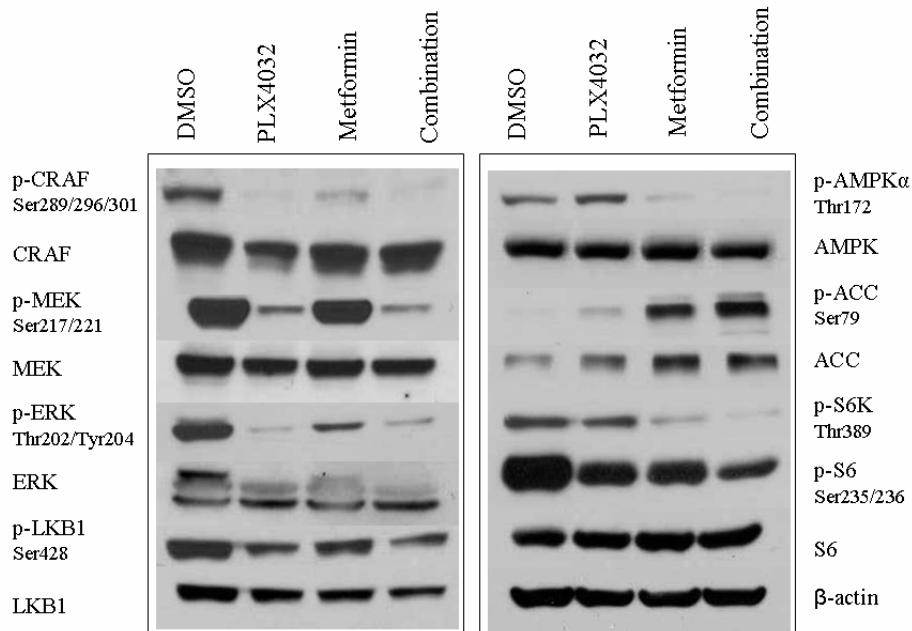

## b. M207 (NRAS mt)

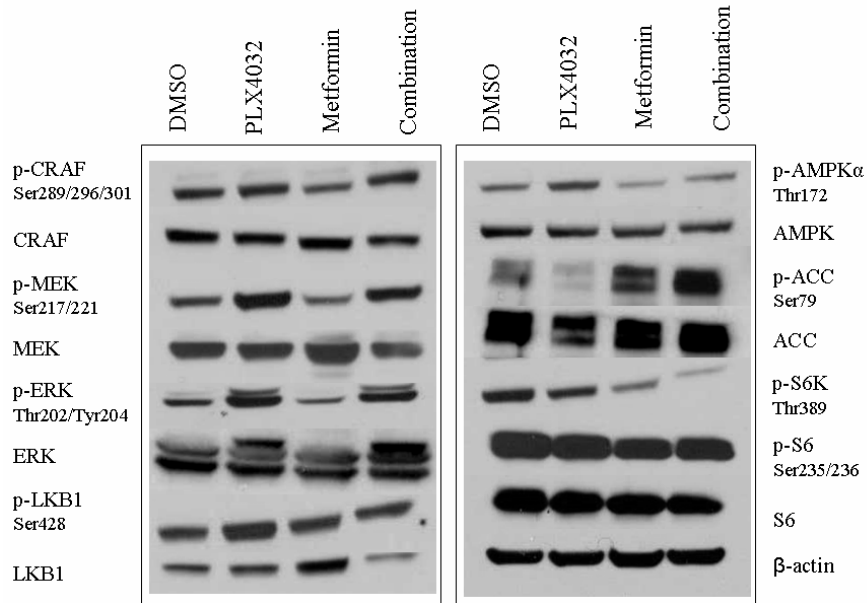

Supplement: Additional file 1 — Western blot analysis of two cell lines with synergy with the combination. To analyze the phospho-protein signaling events triggered by exposure to vemurafenib and metformin, cell lines were treated with DMSO, vemurafenib (5 μM), and metformin (10 mM), either singly or in combination, for 24 hours. Protein phosphorylation was examined by Western Blot analysis and phospho-specific flow cytometry. Blotting for total proteins and β-actin was used as a loading control. Phosphorylation was analyzed with antibodies against pRAF, pMEK, pERK, pLKB1, pAMPKα, pACC, pS6K, pS6, and their total proteins. a) BRAFV600E mutant cell line M263; b) NRASQ61L mutant cell line M207. [file 1479-5876-9-76-S1.PDF]

a. M263 (synergistic)

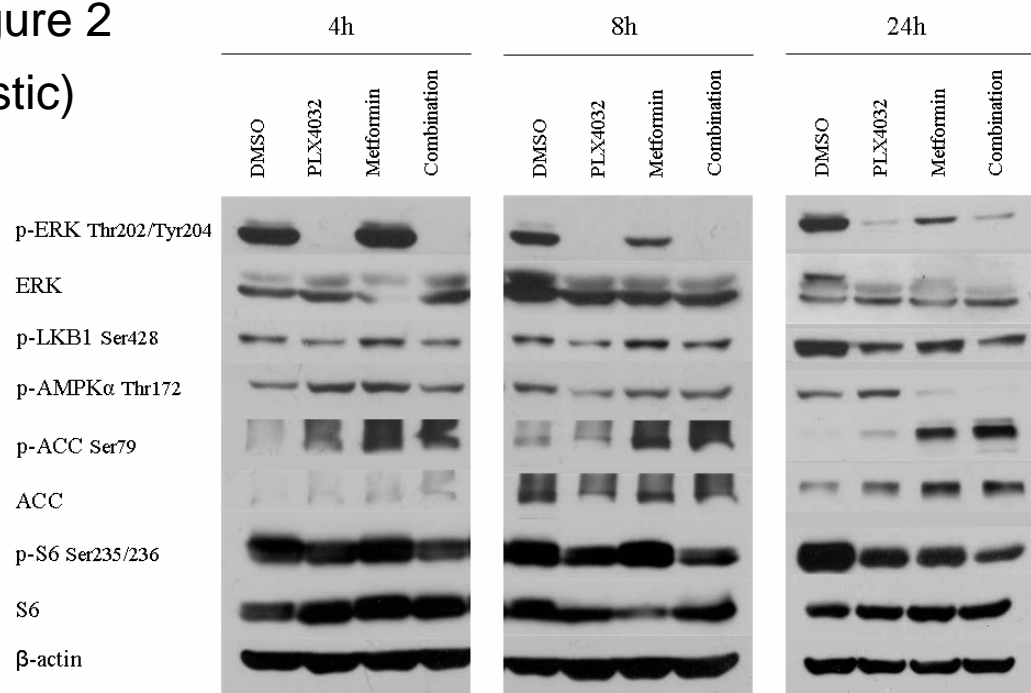

**b. SKMEL28 (antagonistic)**

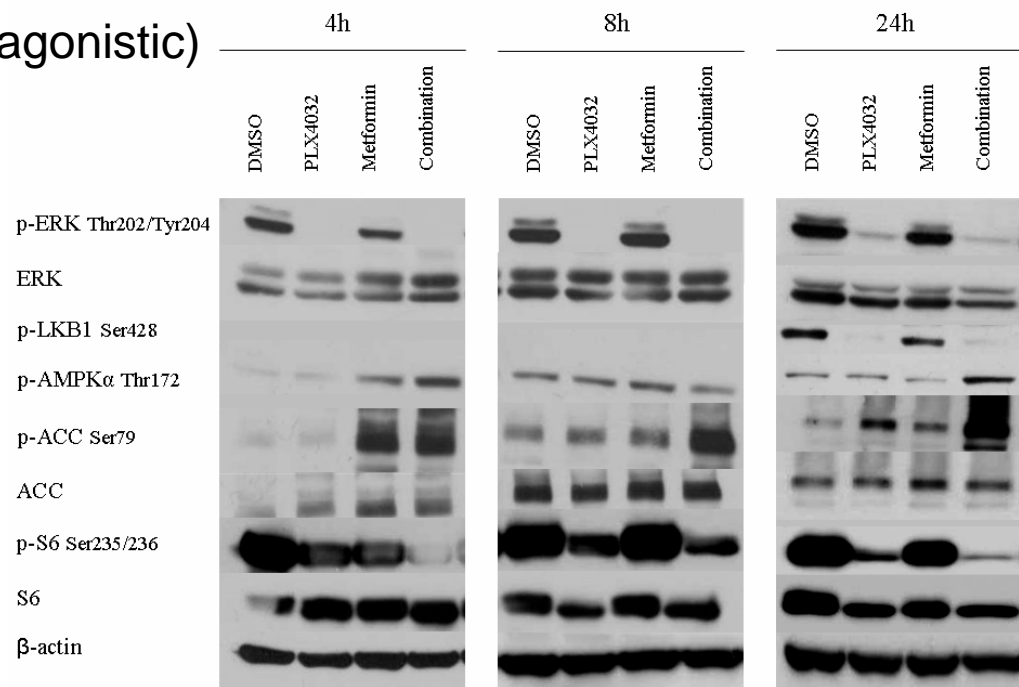

Supplement: Additional file 2 — Time course analysis of signaling in two BRAFV600E mutant cell lines with different response to the combination. Cells were treated for 2, 4, and 24 hours with 5 μM vemurafenib, 10 mM metformin, or the combination. a) M263, in which the combination showed slight synergistic effects in proliferation assays; b) SKMEL28, in which the combination had slight antagonistic effects in proliferation assays. [file 1479-5876-9-76-S2.PDF]

Supplemental Figure 3

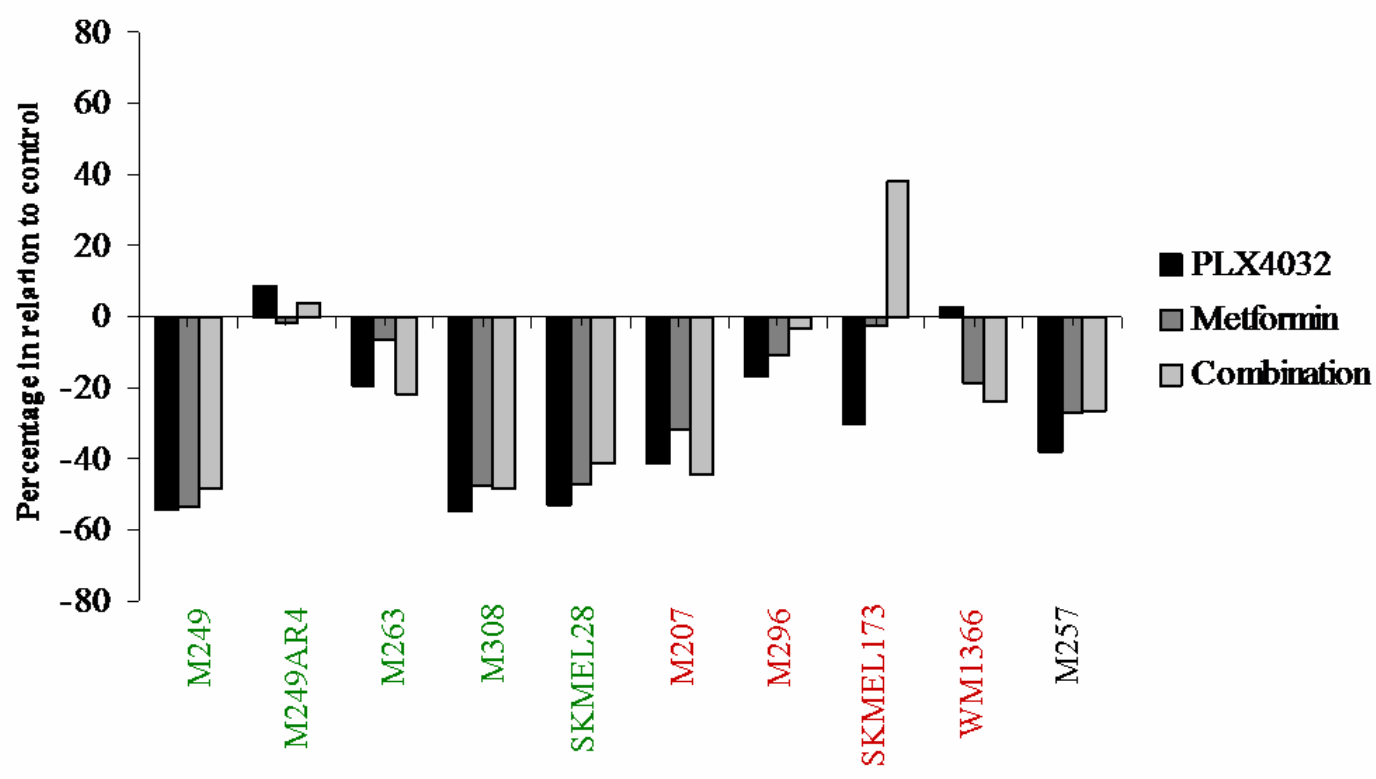

Supplement: Additional file 3 — Phospho-specific flow cytometry for p-Akt Thr308. Cells were treated with vemurafenib (5 μM), metformin (10 mM) or the combination for 24 hours, and intracellularly stained with a pAKT Thr308 antibody. Percentages shown are in relation to DMSO controls and were analyzed by flow cytometry. [file 1479-5876-9-76-S3.PDF]

Supplemental Figure 4

a. M263 (synergistic)

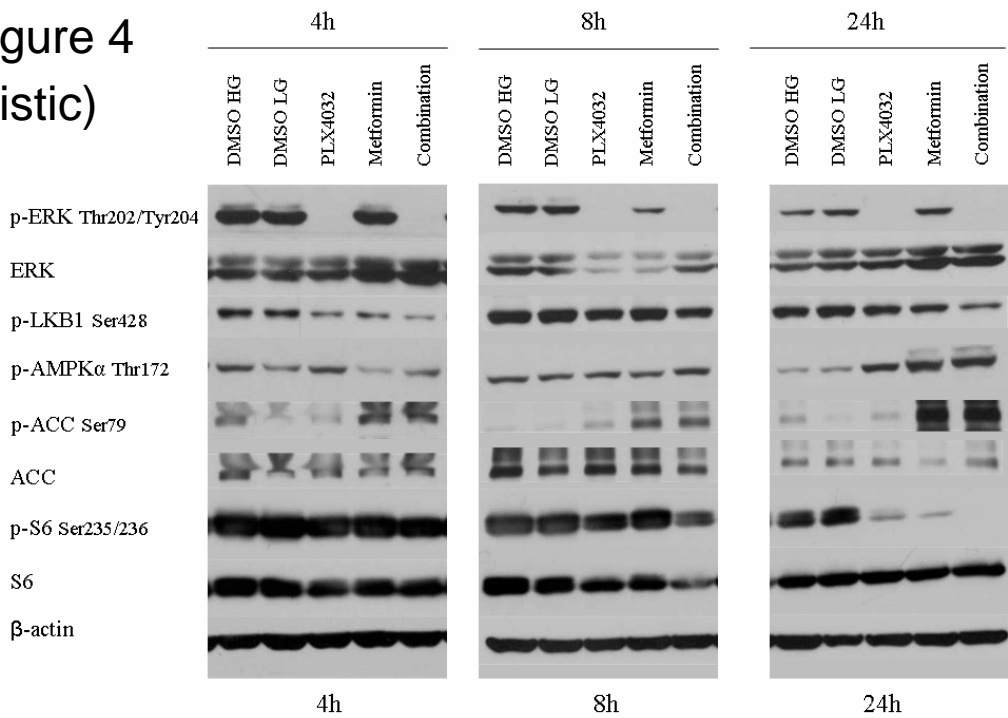

b. SKMEL28 (antagonistic)

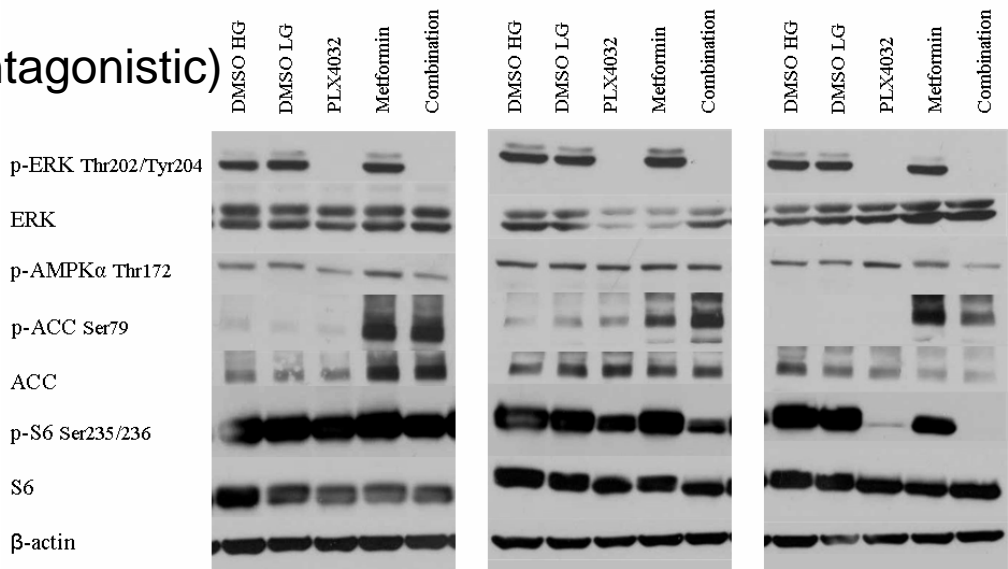

Supplement: Additional file 4 — Time-course Western blot analysis of the BRAFV600E mutant cell lines M263 (top) and SKMEL28 (bottom) in low glucose media. For these studies, the BRAFV600E mutant cell lines M263, resistant to vemurafenib and sensitive to metformin, and SKMEL28, resistant to vemurafenib and metformin, were treated with agents dissolved in low glucose media (1000 mg/L) instead of normal RPMI (3000 mg/L) and analyzed at 4, 8 or 24 hours. [file 1479-5876-9-76-S4.PDF]
